# Sutureless microvascular anastomosis assisted by an expandable shape-memory alloy stent

**DOI:** 10.1371/journal.pone.0181520

**Published:** 2017-07-24

**Authors:** Noriko Saegusa, Shunji Sarukawa, Kunihiro Ohta, Kensuke Takamatsu, Mitsuhiro Watanabe, Takashi Sugino, Masahiro Nakagawa, Yasuto Akiyama, Masatoshi Kusuhara, Kazuo Kishi, Keita Inoue

**Affiliations:** 1 Division of Plastic and Reconstructive Surgery, Shizuoka Cancer Center, Shizuoka, Japan; 2 Department of Plastic Surgery, School of Medicine, Keio University, Tokyo, Japan; 3 Department of Plastic and Reconstructive Surgery, School of Medicine, Jichi Medical University, Tochigi, Japan; 4 Tamachi Industries Co., Ltd, Tokyo, Japan; 5 Takumi Kogyo Co., Ltd, Shizuoka, Japan; 6 Division of Pathology, Shizuoka Cancer Center, Shizuoka, Japan; 7 Research Institute, Shizuoka Cancer Center, Shizuoka, Japan; 8 Avenue Cell Clinic, Tokyo, Japan; Temple University School of Medicine, UNITED STATES

## Abstract

Vascular anastomosis is the highlight of cardiovascular, transplant, and reconstructive surgery, which has long been performed by hand using a needle and suture. However, anastomotic thrombosis occurs in approximately 0.5–10% of cases, which can cause serious complications. To improve the surgical outcomes, attempts to develop devices for vascular anastomosis have been made, but they have had limitations in handling, cost, patency rate, and strength at the anastomotic site. Recently, indwelling metal stents have been greatly improved with precise laser metalwork through programming technology. In the present study, we designed a bare metal stent, Microstent, that was constructed by laser machining of a shape-memory alloy, NiTi. An end-to-end microvascular anastomosis was performed in SD rats by placing the Microstent at the anastomotic site and gluing the junction. The operation time for the anastomosis was significantly shortened using Microstent. Thrombus formation, patency rate, and blood vessel strength in the Microstent anastomosis were superior or comparable to hand-sewn anastomosis. The results demonstrated the safety and effectiveness, as well as the operability, of the new method, suggesting its great benefit for surgeons by simplifying the technique for microvascular anastomosis.

## Introduction

Successful vascular anastomosis is the highlight of cardiovascular and transplant surgery, which is currently performed by hand using a needle and suture. The history of this technique is surprisingly long, going all the way back to 1902, when Alexis Carrel first reported it as a practical way to approximate vessel ends, minimizing the gap between their lumens [1]. This has made a great impact on modern surgery that enables all sorts of procedures that require connecting blood vessels to vessels. In the 1960s, concurrently with the development of surgical microscopes, anastomosis of vessels with a diameter smaller than 2–3 mm was achieved [2]. That pioneered the way to ‘microvascular’ anastomosis, by which free flap transfer, digit replantation, and coronary artery bypass grafting became possible.

The most crucial objective of microvascular anastomosis is supplying fresh blood to the organs downstream of the anastomosis as quickly as possible. For a surgeon to acquire quick and accurate anastomosis technique, a certain amount of specialized training is required. However, even with skilled surgeons, anastomotic thrombosis occurs in approximately 0.5–10% of the cases. Moreover, repeat surgery is extremely difficult when complications occur, and tissue loss can occur in the worst-case scenario. Therefore, to reduce the burden on the surgeons through a simplified procedure that does not require specialized technique and to improve the surgical outcome, research and development of a device that facilitates microvascular anastomosis are needed.

The development of automatic vascular anastomosis instruments began around 1900, and techniques and materials such as vascular stapling and clipping [3–5], ring pins [6,7], tubes and stents [8,9], adhesives [10], welding [11], and soluble polymer stents have been used [12]. However, compared to hand-sewn anastomosis, there are some limitations, such as difficulty in handling the walls of atherosclerotic vessels, difficulty in joining vessels with mismatched diameters, high cost, low patency rate, and poor anastomotic strength. For these reasons, such instruments are rarely actually used [13].

In the cardiovascular field, vascular indwelling metal stents are widely used in the form of bare-metal stents (BMSs) and drug-eluting stents (DESs) for percutaneous coronary intervention (PCI). These stents for PCI are known to achieve a certain level of successful outcomes with respect to the biological safety of their placement and their patency rates [14]. Furthermore, with the recent improvement in intricate metal processing technology that uses laser and electrolytic polishing technology, the creation of a novel, delicate, yet strong stent has become feasible. Using these technologies, we developed a BMS, termed Microstent, constructed with NiTi, a shape-memory alloy (SMA), with a design and size that do not interfere with blood flow such that it can be placed in the microvascular lumen. We then devised a technique for sutureless anastomosis by placing a Microstent at the end-to-end anastomotic site and gluing the surrounding areas. In the present study, this technique was performed in rats, and ex vivo strength tests were conducted in order to verify the safety and effectiveness, as well as operability, of the newly developed stent.

## Materials and methods

### Animals

Pathogen-free, 13-week-old, male SD rats were purchased from Japan SLC Inc. (Shizuoka, Japan) and housed humanely according to guidelines for the welfare and use of animals in cancer research published in the British Journal of Cancer in 2010 and procedures approved by the Animal Care and Use Committee of Shizuoka Cancer Center Research Institute. We visually monitored the animals once a day. If any animal showed decreased activity it was physically checked. The procedure for severely sick rats was euthanasia using inhalation anesthesia with isoflurane, same as the endpoint.

### Animal model and surgical techniques

The rats were anesthetized using inhalation anesthesia with isoflurane, and all efforts were made to minimize suffering. The ventral surface was shaved with electric clippers, and a midline laparotomy incision was made. The infrarenal aorta was isolated under a surgical microscope (MEIJI TECHNO Inc., Tokyo, Japan). The rats were injected with heparin sodium 100 IU/kg and acetylsalicylic acid 5 mg/kg before aortic clamping to decrease the risk of aortic thrombosis. Two mg of papaverine hydrochloride were sprinkled over the surgical field to control vasospasm. After clamping the aorta with microvascular clamps (S&T AG, Neuhausen, Switzerland), the aorta was divided, and the lumen was irrigated with heparinized saline. Both clamps were repositioned so that the vessels completely covered the Microstent. The Microstent was compressed with 7–0 nylon thread that was inserted into each end. When the thread was removed, the stent expanded, and cyanoacrylate glue (Aron alpha A, Sankyo Inc., Tokyo, Japan) was applied around the connected ends of the vessels. After the connection adhered, the clamps were released, and blood patency was checked (n = 46). The midline incision was closed with 5–0 nylon sutures (Ethicon-Johnson & Johnson Japan, Tokyo, Japan). In the hand-sewn group (n = 43), both ends of the infrarenal aorta were anastomosed with 10–0 nylon thread (KEISEI Medical Industrial, Tokyo, Japan).

### Histology

Anastomosed vessels were harvested at 2 weeks (n = 4 per group), 7 weeks (n = 4 per group), and 26 weeks (n = 4 per group) after the operations and fixed with 4% formalin. After the vessels were placed in acrylic resin blocks, the samples were sectioned into 5-μm-thick slices. The samples were stained with H&E or elastic Van Gieson stain (Sept Sapie Inc., Tokyo, Japan). A virtual slide system, NanoZoomer and NDP.view (Hamamatsu, Hamamatsu, Japan), was used to observe and measure sections.

### Imaging studies

The vessel diameter, flow, the existence of thrombosis, and morphological changes at one week after the operations of all Microstent groups (n = 46) were evaluated using ultrasonography (Dermacup, Atys Medical, Soucieu-en-Jarrest, France) and Doppler sonography (ES-100V3, Hadeco, Tokyo, Japan). Scanning electron microscopy of the lumen of the anastomosed vessels was performed at 2 weeks (n = 1 Microstent group), 7 weeks (n = 1 per group), and 26 weeks (n = 1 per group) after the operations.

### Stress tests on anastomosed vessels

The compliance of anastomosed vessel specimens that were excised from the rats was evaluated in terms of blood flow and pressure changes at 2 weeks (n = 6 hand-sewn group, n = 7 Microstent group), 7 weeks (n = 6 per group), and 26 weeks (n = 6 per group) using a custom-designed device (LMS Inc., Tokyo, Japan) for an ex vivo circulation system, consisting of a pulsatile cardiac pump (ALPHA FLOW EC-1, Fuyo Corporation, Tokyo, Japan), outlet flow meter (FD-SF1, KEYENCE Japan, Osaka, Japan), data-acquisition system (Labscribe, iWorx System Inc., Dover, NH), CCD camera (Shodensha, Osaka, Japan), and a light source (LMS Inc., Tokyo, Japan). The burst-strength and tensile strength of the specimens were also tested using a custom-designed device (LMS Inc., Tokyo, Japan). The burst-strength at 2 weeks (n = 3 each group), 7 weeks (n = 3 per group), and 26 weeks (n = 3 per group), and the tensile strength at 2 weeks (n = 6 hand-sewn group, n = 7 Microstent group), 7 weeks (n = 6 per group), and 26 weeks (n = 5 hand-sewn group, n = 6 Microstent group) were each evaluated.

### Statistical analysis

All statistics were performed using Microsoft Excel software. The operation times (ischemic time) for hand-sewn and Microstent anastomoses were compared by Student’s *t*-test. The incidence of thrombosis and undefined rat death were compared by Fisher’s exact test. Tensile strength of ex vivo samples was compared by Student’s *t*-test. Numerical data of histology, including lumen diameter and tunica media and intima thicknesses, were averaged and compared by Student’s *t*-test. Student’s *t*-test was chosen because the sample size was small. P < 0.05 was considered significant.

## Results

### Designing and laser machining of the NiTi Microstent

**Fig 1:**
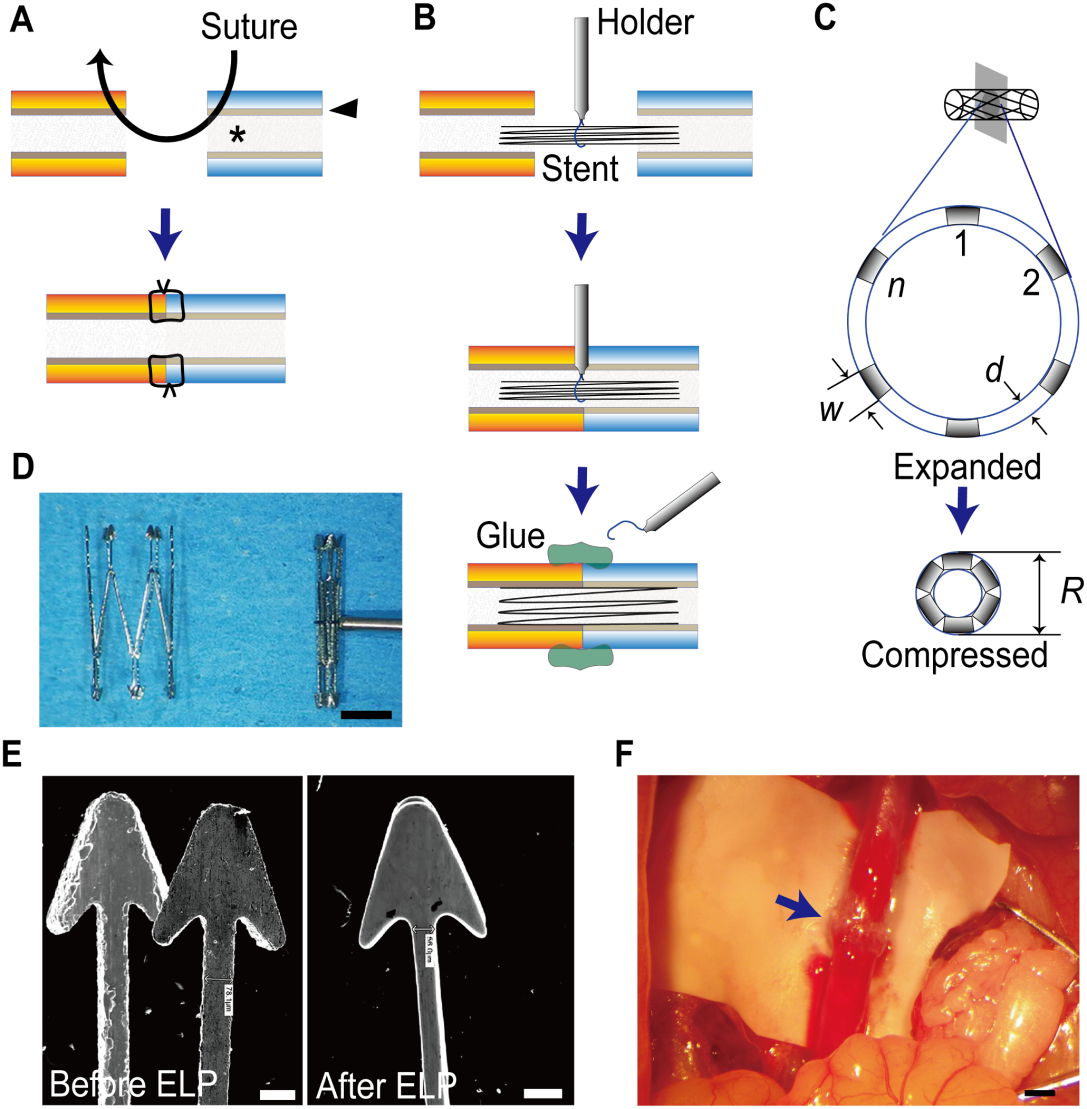
Stent-assisted sutureless microvascular anastomoses. (**A**) Showing the traditional microvascular anastomosis using a needle and thread. Multiple interrupted sutures, usually 6–10, are required for anastomosis. The key to success is to maximize the vessel lumen (asterisk) by coaptation of both ends of the intima (arrowhead) without a gap and placing sutures at even intervals. (**B**) The Microstent system to perform sutureless microvascular anastomosis. The Microstent compressed by a 7–0 nylon thread squeezed in the holder is inserted into each side of the vessel ends (top). After both ends are coapted (center), the 7–0 nylon thread is cut to loosen the Microstent, and the holder is removed. The Microstent automatically expands to maintain the lumen without a gap (bottom). Finally, cyanoacrylate glue (green) is applied around the junction of the vessels (See also S1 Movie). (**C**) The approximate diameter (*R*) of the compressed Microstent is determined by the number of branches (*n*), tube thickness (*d*), and branch width (***w***). When the Microstent is compressed to the maximum extent, the branches touch each other. (**D**) NiTi stent (Microstent) fully expanded (2 x 4 mm) (left) and a stent compressed by a 7–0 nylon thread (right). Note the continuously arranged branches in a Z-shape at the center in order to be compressed uniformly end to end. Bar: 1 mm. (**E**) Scanning electron microscope image of the Microstent before and after electrolytic polishing (ELP), Bars: 100 μm. (**F**) Intraoperative view of anastomosis of rat aorta using Microstent. The anastomotic site was sealed by cyanoacrylate (arrow). Bar: 1 mm.

The Microstent was designed to be initially compressed at the center with a nylon thread, and then inserted from the end of the blood vessels at the anastomosis site. Once the nylon thread was loosened, it automatically restored its shape to expand the lumen (Fig 1A and 1B). The metal to be placed in the blood vessel must have high biocompatibility. We therefore chose NiTi, an SMA, as the material for the Microstent, a material that had already been used in coronary artery stents and other types of vascular stents. In addition, the design must be such that it has minimal unevenness and reduces the impact on blood flow at the anastomosis site. Hydrodynamic simulation showed that thickness was the primary factor that affects blood flow stagnation. Blood flow stagnation was particularly large at the junction between the branches, and because a markedly greater decrease in the area where stagnation occurs was observed with a thickness of 100 μm than with 150 μm (S1 Fig), a NiTi tube with a thickness of 100 μm was used. When the inner diameter of the microvessel to be anastomosed is approximately 2 mm under conditions in which blood flow is present, it contracts to approximately 1.2 mm under conditions in which the blood vessel is clamped. In order to insert the stent smoothly, it was necessary for the stent to have a design such that its size prior to expansion was ≤ 1.2 mm (Fig 1C). The diameter *R* of the stent before expansion is dependent on the width (*w*), thickness (*d*), and number (*n*) of stent branches. When ideal compression is attained, the theoretical *R* is defined as follows:

R=wn/π+2d

To reach a compressed diameter of *R* < 1.2 mm, the upper limit of branch width *w* is < 0.262 mm when *d* = 0.1 mm and *n* = 12, and < 0.523 mm when *d* = 0.1 mm and *n* = 6. Through trial and error, we ultimately created a Microstent designed with six grips (width 0.3 mm) at both ends and twelve branches (width 0.1 mm) continuously arranged in a Z-shape at the center (Fig 1D). Based on this design, NiTi tubes 1 mm in diameter and 0.1 mm in thickness were manufactured with a laser welding machine (ROFIN-SINAR Technologies Inc., Plymouth, MI) using laser programming technology, and shape memory was subsequently created through heat treatment such that the diameter would be 2 mm in the expanded state. Electrolytic polishing was performed such that the stent had adequately small surface roughness (0.1%) (Fig 1E). As a result, the size of the Microstent was 2 mm (diameter) x 4 mm (length) in its fully expanded state. When compressed, the diameter contracted uniformly from end to end to 0.8 mm. The branch was ≤ 60 μm at its thinnest part. Although this stent was narrower and thinner than a standard coronary stent, it had sufficient radial force suitable for operation.

### Feasibility of the Microstent: Operation time and postoperative thrombosis

Next, in order to evaluate the feasibility in actual vascular anastomosis, we used the infrarenal aortas of Sprague-Dawley (SD) rats, which have similar characteristics to peripheral arteries in humans. The Microstent anastomosis technique was as follows. 1. After clamping the infrarenal aorta and cutting it apart, the Microstent was compressed by a 7–0 nylon thread that was inserted into each end of the vessels. 2. After the vessels were approximated, the Microstent was expanded by removing the 7–0 nylon thread. 3. Cyanoacrylate glue was applied around the connected ends of the vessels followed by declamping. (Fig 1B and S1 Movie). The Microstent was evenly compressed from the center to both ends, and it could be inserted smoothly into the ends of the blood vessels. By releasing the nylon thread, the Microstent immediately expanded and pushed the lumen from the inside. The Microstent was fixed in this state. One plastic surgery specialist performed all hand-sewn anastomoses and all Microstent anastomoses to facilitate comparison of the two groups. Eighty-eight rat (hand-sewn group, n = 43, Microstent group, n = 45) were sacrificed at the endpoint. Nine rats in the hand-sewn group died prior to endpoint, three rat died from overdosed anesthesia, three rats that became ill from unknown causes were euthanized, two died from thrombosis, and one from injury of the urinary tract. In the Microstent group, five rats died before endpoint. Two rats died from overdosed anesthesia, one rat that became ill from unknown causes were euthanized, one died from a renal disease, and one from a pseudoaneurysm.

**Fig 2:**
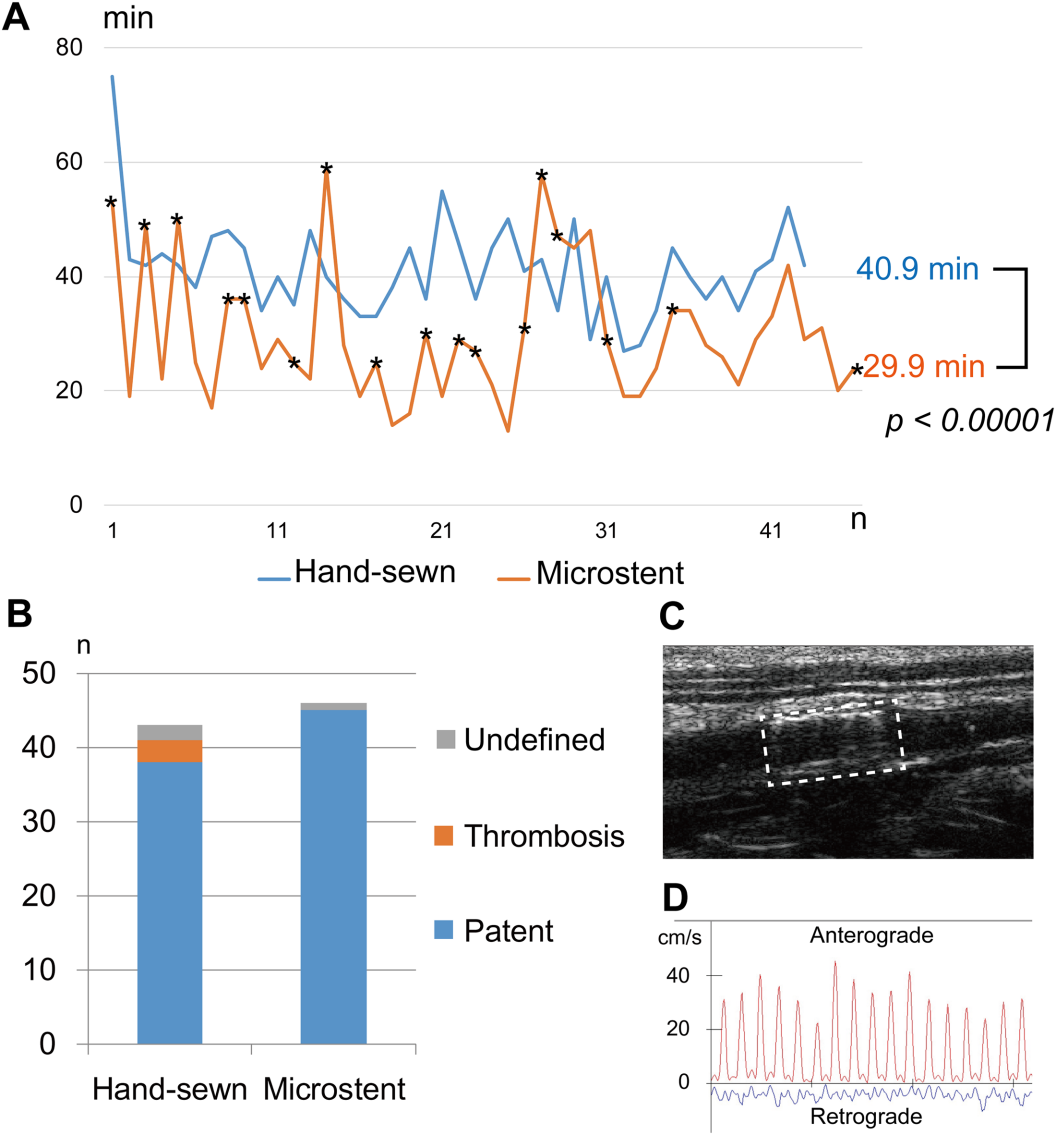
Operation time and postoperative thrombosis in Microstent anastomosis. (**A**) Operation time of microvascular anastomoses in rats. A single surgeon performed anastomosis of the infrarenal aorta of the rat by 10–0 nylon suture and needle (*n* = 43) (blue) or by Microstent (*n* = 45) (orange). The lines demonstrate the changes in the ischemic time of the infrarenal aorta during anastomosis with the number of operations. The operation time for the Microstent anastomosis includes time for gluing by cyanoacrylate as well. The average ischemic time of the hand-sewn group was 40.9 min, while the average time of the Microstent group was 29.9 min. In some cases, the first operation to implant the Microstent failed, and a second or third one was required. In such cases, the ischemic time was prolonged (asterisks). There was a significant difference between the two groups (*p* < 0.00001). *p* values were determined by Student’s *t*-test. (**B**) The bar chart shows evidence of thrombosis a week after the operations (orange). Some of the rats died due to undefined reasons without anastomotic thrombosis (grey). The thrombosis rate was 2.3% in the hand-sewn group. No significant difference in the thrombosis rate was found between the hand-sewn group and the Microstent group (*p = 0*.*104)*. *p* values were determined by Fisher’s exact test. (**C**) Ultrasonography of an implanted Microstent (dashed square). The Microstent is visualized as a high-echoic region inside the aortic lumen (see also S2 Movie). (**D**) Doppler studies of the peripheral aorta to the anastomotic site demonstrate patent blood flow a week after operation in the Microstent group.

The average ischemic time, which is the time from aorta clamping to declamping, to perform Microstent anastomosis was 29.9 min (range 14 min to 59 min). This was significantly shorter than the ischemic time required for hand-sewn anastomosis (40.9 min (range 29 min to 75 min)) (*p* < 0.00001) (Fig 2A). The reason why a longer duration was sometimes required with the Microstent was because the insertion was repeated due to Microstent dislodgement at the first trial, or because the procedure was repeated due to hemorrhage caused by failing to apply the adhesive. Nonetheless, the operation could be completed in all cases.

The postoperative formation of thrombosis at the anastomotic site in the aorta caused direct death of the animals. By the analyses of dead animals, the rate of postoperative anastomotic thrombosis was found to be 2.3% in the hand-sewn group, whereas no thrombosis occurred in the Microstent group (*p* = 0.104) (Fig 2B). Doppler ultrasonography to detect blood flow through the anastomotic site also showed no signs of thrombosis in live animals one week after the operations (Fig 2C and 2D and S2 Movie). There were some deaths due to complications other than anastomotic thrombosis, such as overdosed anesthesia or infection in both groups prior to the endpoint, although the numbers were very small (Fig 2B). There were two cases of pseudoaneurysms, one developed prior to the endpoint and one revealed at the endpoint, most likely because both ends of the aorta did not approximate during stent expansion because a sufficient length could not be attained at the site of the blood vessel for stent insertion due to vasoconstriction after severing the rat infrarenal abdominal aorta. However, there was no evidence of intraluminal thrombosis or occlusive lesions in the Microstent group.

### Wall compliance and mechanical strength of Microstent-anastomosed vessels

**Fig 3:**
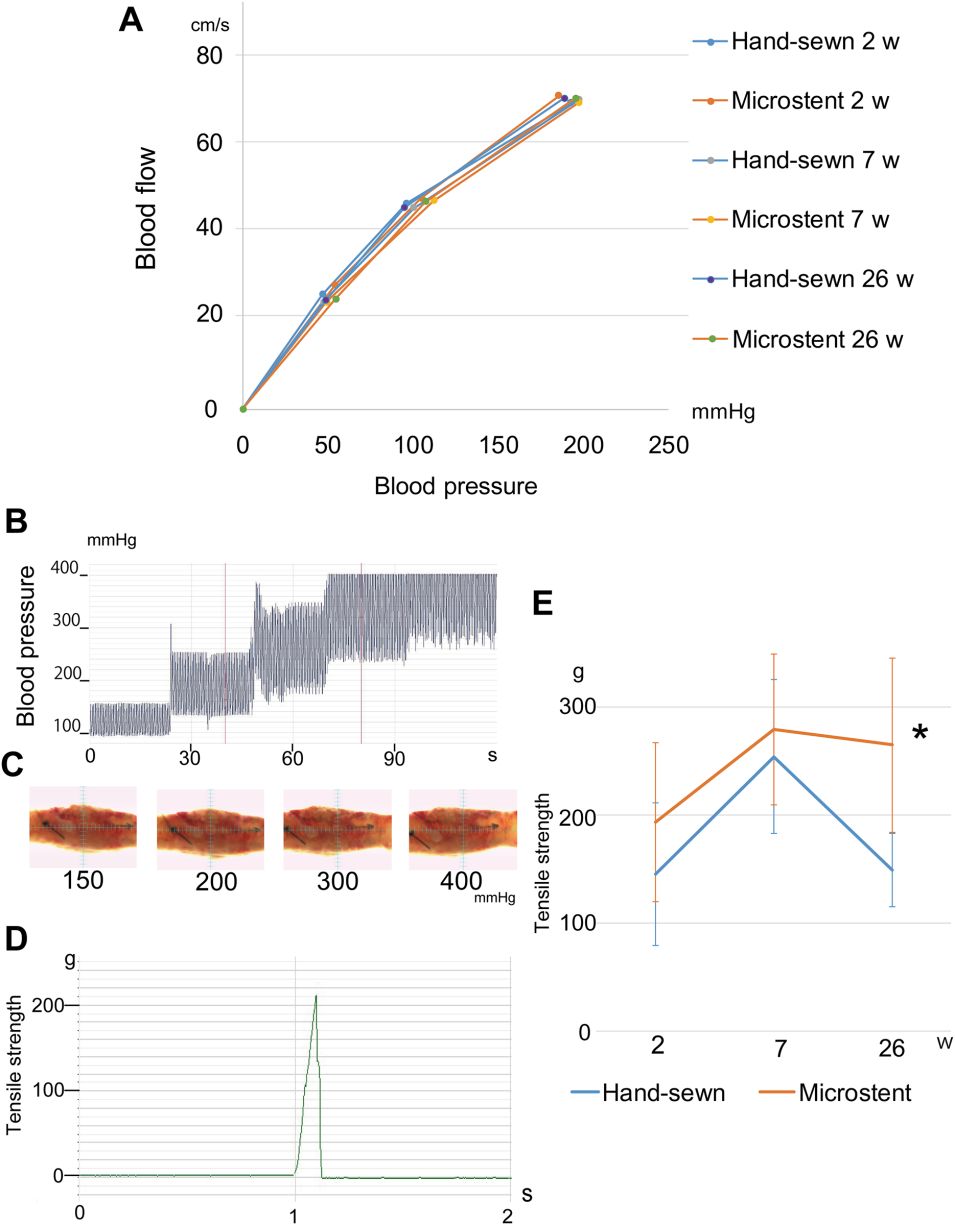
Ex vivo analyses of wall compliance and mechanical strength of Microstent anastomosed vessels. (**A**) The anastomosed vessels are connected to an ex vivo cardiac pump system. The lines demonstrate the average change in blood pressure with the blood flow change of the vessel specimen at 2 weeks, 7 weeks, and 26 weeks in each group (n = 6). (**B**) The representative data of the pressure changes of the Microstent-anastomosed vessel under cardiac pump pulsation. Both groups could withstand the highest arterial pressure of over 400 mmHg (see also S3 and S4 Movies). (**C**) Photograph of Microstent-anastomosed vessels under different arterial pressures: 150, 200, 300, and 400 mmHg. (**D**) The representative tensile curve of the anastomosed vessel. The vessels are pulled under physiological pulsation and blood pressure (see also S5 and S6 Movies). (**E**) The average tensile strength of hand-sewn and Microstent-anastomosed vessels at 2, 7, and 26 weeks. There is a significant difference between the two groups at 26 weeks. *p* values were determined by Student’s *t*-test. *p < 0.*05*.

In ex vivo specimens, whether the Microstent anastomosis had sufficient mechanical strength, vessel status with blood pressure changes, and subsequent vessel wall deformity were examined. By connecting the specimens to an ex vivo cardiac pump system, the relationships between blood flow and blood pressure changes were explored at postoperative 2 weeks (n = 6 hand-sewn group, n = 7 Microstent group), 7 weeks (n = 6 per group), and 26 weeks (n = 6 per group). The blood pressure shift with the blood flow change showed no significant differences at 2 weeks, 7 weeks, and 26 weeks in each group, suggesting that their wall compliances were similar (Fig 3A).

Burst-strength was also examined at 2 weeks (n = 3 each group), 7 weeks (n = 3 per group), and 26 weeks (n = 3 per group). Both groups could withstand arterial pressure over 400 mmHg (Fig 3B and 3C, S3 and S4 Movies). Tensile tests at 2 weeks (n = 6 hand-sewn group, n = 7 Microstent group), 7 weeks (n = 6 per group), and 26 weeks (n = 5 hand-sewn group, n = 6 Microstent group) demonstrated that the average tensile strength of the Microstent group was stronger than that of the hand-sewn group at all weeks (significantly at 26 weeks, *p* < 0.05) (Fig 3D and 3E, S5 and S6 Movies).

### In vivo biocompatibility of the Microstent

**Fig 4:**
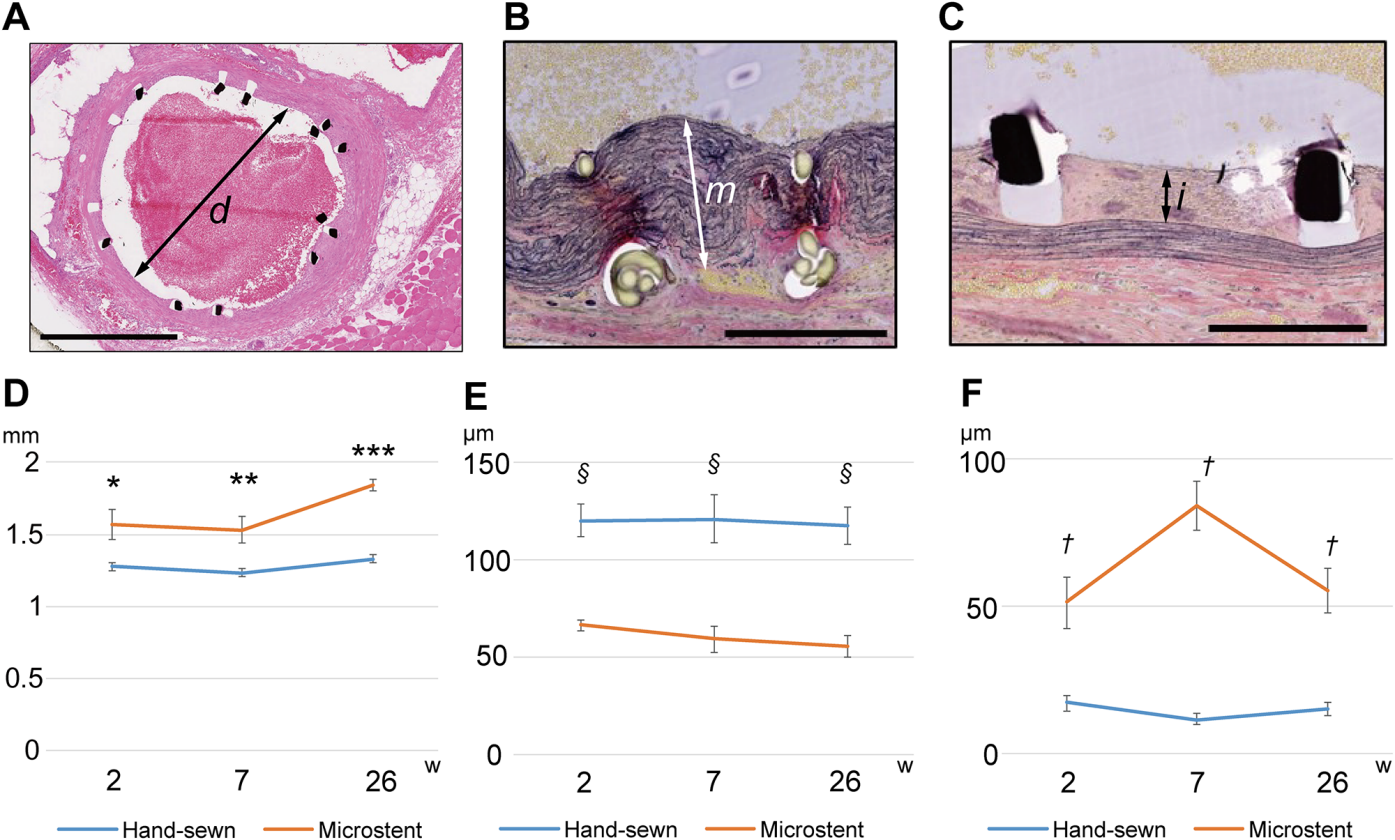
Histological change in the anastomosed vessels. (**A**) A cross-section of a Microstent-anastomosed vessel at 7 weeks (H&E stain). The internal diameter (*d*) of the lumen is measured in 4 different directions and averaged. Bar, 1 mm. (**B**) A magnified view of a hand-sewn vessel wall at 7 weeks. Elastic Van Gieson stain. The thickness of the tunica media (*m*) in the middle of the sutures or the stent branches is measured. Bar, 250 μm. (**C**) Neointimal cell proliferation in a Microstent-anastomosed vessel at 7 weeks. Elastic Van Gieson stain. The thickness of the intima (*i*) in the middle of the sutures or the stent branches is measured. Bar, 250 μm. (**D**) Internal diameter at 2 weeks, 7 weeks, and 26 weeks. No significant differences are found between the hand-sewn group and the Microstent group. *p = 0.*106*, **p = 0.*058*, ***p = 0.*061*. (**E**) Thickness of the tunica media of each group at 2 weeks, 7 weeks, and 26 weeks. The tunica media is thicker in the hand-sewn aortas than in the Microstent-anastomosed aortas. There is a significant difference between the two groups. *p* values were determined by Student’s *t*-test. § *p < 0*.*05*. (**F**) Neointimal cell proliferation of each group at 2 weeks, 7 weeks, and 26 weeks. Neointimal cell proliferation of the Microstent-anastomosed aortas is more active than that of the hand-sewn aortas. There is a significant difference between the two groups. P values were determined by Student’s *t*-test. †*p < 0*.*05*.

To evaluate the acute and chronic histological changes of the anastomotic site after Microstent implantation, anastomosed vessels were harvested at 2 weeks (n = 4 per group), 7 weeks (n = 4 per group), and 26 weeks (n = 4 per group). The internal diameter of the Microstent group was larger than that of the hand-sewn group at every time point, although there were no significant differences between the groups (Fig 4A). The tunica media of the hand-sewn aortas was significantly thicker than that of the Microstent-anastomosed aortas (*p* < 0.05) (Fig 4B). Conversely, while vascular endothelial cell proliferation was observed with the usage of the Microstent, similar to that seen with cardiovascular stents, it decreased at 26 weeks. Moreover, the inner diameter of the blood vessels was maintained up to 26 weeks due to the shape-memory function of the NiTi in the Microstent, and stenosis was not observed. Neointimal cell proliferation was significantly more active in the Microstent-anastomosed aortas than in the hand-sewn aortas at all time points (*p* < 0.05) (Fig 4C).

**Fig 5:**
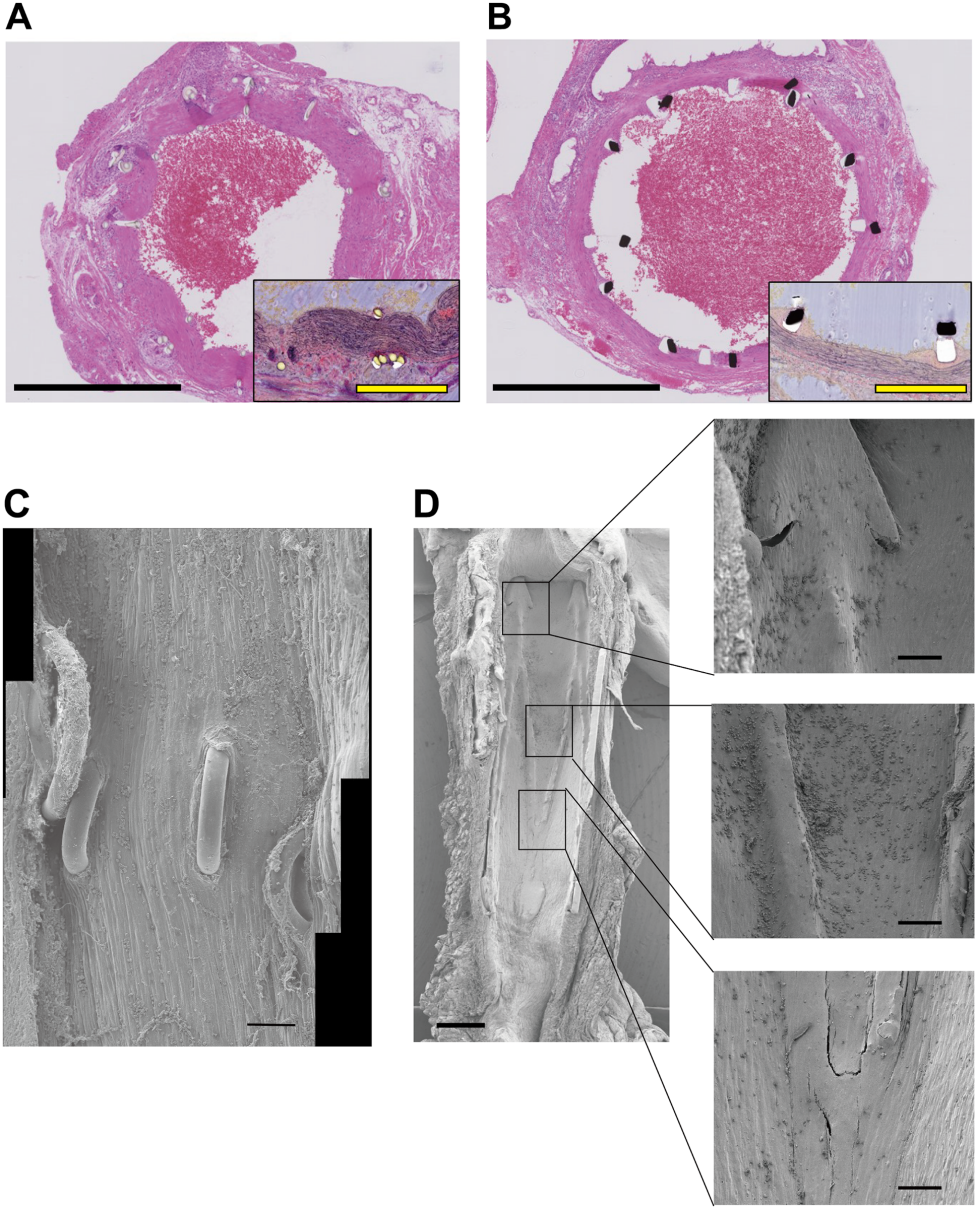
Histological examination of anastomosed vessels. (**A**) A cross-section of a hand-sewn rat infrarenal aorta at 26 weeks. Low-magnification view (H&E stain). High-magnification view (elastic Van Gieson stain) shows hyperplasia of the tunica media. Black bar, 1 mm; yellow bar, 250 μm. (**B**) A cross-section of a Microstent-anastomosed rat infrarenal aorta at 26 weeks. High-magnification view shows neointimal proliferation limited to the region around the stent branches. (**C**) A SEM view of the lumen of a hand-sewn vessel at 26 weeks showing thread exposure into the lumen. Bar, 200 μm. (**D**) A SEM view of the lumen of a Microstent-anastomosed vessel at 26 weeks. The vessel is half-cut. All branches of the Microstent are covered with endothelium. Left bar, 500 μm; right bars, 100 μm.

The photomicrographs of aortas stained with H&E and elastic Van Gieson stains at 26 weeks clearly showed the changes of the tunica media and the endothelium (Fig 5A and 5B). Scanning electron microscopy (SEM) of the vessel lumen at postoperative 2 weeks (n = 1 Microstent group), 7 weeks (n = 1 per group), and 26 weeks (n = 1 per group) showed that, in the hand-sewn group, threads were still exposed in the lumen at 7 weeks and 26 weeks after surgery (Fig 5C). On the other hand, all aspects of the stems of the Microstent were completely covered by endothelium at that latest at postoperative 26 weeks (Fig 5D).

## Discussion

There have been previous attempts to maintain the vascular lumen through the insertion of some devices to assist sutureless anastomosis [12,13]. Nonetheless, there is still no such device in clinical use. Although the use of metal stents has been described previously, these have not been used due to their low patency rates and arteriovenous fistula development [8]. However, the technological innovation of metal stents used in PCI is remarkable, and the Microstent has been re-evaluated with the improved precision in laser metalworking through programming technology. It has become feasible through this technological innovation to design a stent with branch and grip sizes that are calculated at the micrometer level. In the present study, the creation of a vascular anastomosis stent that is unprecedented in its small size (contracted diameter of 0.8 mm) was achieved.

Since coronary stents and vascular stents have a closed-cell configuration and cannot be inserted into the lumens of vessel ends because both ends of the stent will flare open if the center is tied. In contrast, both ends of the Microstent could be compressed evenly because the branch is composed solely of symmetrical Z-shaped open cells, and this led to successful and straightforward stent insertion. Although there are previous experimental reports of using stents for anastomosis, the procedure requires the closed-cell stent to be placed at the anastomosis site through a catheter from a different location than the anastomosis site and the catheter to be subsequently withdrawn for the expansion of the SUS stent with a separate unit such as a balloon [15], indicating poor usability and feasibility, in that an extra catheter maneuver is necessary. In addition, excess branches are unnecessary in stents for anastomosis, unlike coronary stents whose purpose is to expand the site of stenosis. Therefore, the Microstent is constructed with the minimum width and the minimum number of grips (six) along with their corresponding branches (twelve). An excessive number of wide branches, as seen in previous stents for anastomosis, only triggers unnecessary thrombus formation. We therefore believe that stents with open zigzag cells such as the Microstent will become the future standard of anastomotic stents.

The Microstent group had significantly shorter intraoperative ischemic time compared to the hand-sewn group. Although ischemic time could not be reduced to less than 20 min with hand-sewn anastomosis even with over 40 sessions of training, the ischemic time could be shortened to less than 20 min with the Microstent with the same number of training sessions. By using the Microstent, it is possible to perform microvascular anastomosis, which typically requires at least one year of training and specialized technique, in an intuitive manner for self-learning and in a short amount of time. This consequently expands medical care using microvascular anastomosis, ultimately bringing tremendous benefits to the patients.

Since the Microstent procedure is a non-suture anastomosis that only uses adhesives, there was concern that the mechanical strength would be inferior. However, vascular evaluations by an ex vivo cardiac pump system showed that the Microstent had similar or better mechanical characteristics compared to hand-sewn anastomosis. Achieving similar or significantly better outcomes in tensile strength and pressure tests indicates that the Microstent can be functionally used in microvascular anastomosis similarly to or more effectively than hand-sewn anastomosis. For the mechanical strength of the anastomosed vessel, the healing of the vessel wall tissue itself through the repair mechanism is important, and the present results were consistent with the report that the existence of sutures is mechanically irrelevant [12].

Histopathological examination showed mild local intimal thickening similar to that seen when cardiac stents are used, but not to an extent that the inner diameter changed. It is known that, in the case of PCI using a bare-metal stent, neointimal cell proliferation arises from migration and proliferation of vascular smooth muscle cells, and in-stent restenosis emerges in approximately 20–30% of cases [16]. This is postulated to occur because the stems are tightly arranged in coronary stents, a greater proportion of the intimal area is in contact with the metal, and the combination of these leads to narrowing of the inner diameter. In contrast, because the Microstent has fewer stems and a narrow and thin structure, there were minimal changes in the intima at the interstem region, although intimal thickening was observed on both sides of the metal. The current trend for managing a narrowed lumen caused by intimal thickening is to consider surface processing, such as drug coating [17]. However, it was possible to minimize the intimal thickening by designing a narrow vascular anastomosis stent consisting of fewer stems. This method could become a novel and crucial strategy to prevent in-stent restenosis. Since the biological responses to NiTi may differ between humans and animals such as rats, further careful observation with experiments using large animals such as pigs is understandably necessary to achieve clinical application.

In PCI, antiplatelet agents must be taken long-term after stent insertion to prevent blood clots. In the present study, thrombus formation was prevented in the Microstent group with a single intravenous administration of heparin and acetylsalicylic acid [18]. Thrombus formation initiates when thrombogenic substances (such as tissue factors and collagen) exposed at the site of injury and a gap on the intima come into contact with platelets. Blood retention is also known to promote thrombus formation. There was evidence of postoperative anastomotic thrombosis in the hand-sewn group, but not in the Microstent group. Electron microscopy showed the presence of exposed nylon thread in the blood vessel with the hand-sewn method at 26 weeks postoperatively. In contrast, the Microstent was completely covered in endothelium and the lumen was smooth, indicating that the Microstent creates an environment that is less suitable for thrombus formation to occur compared to the hand-sewn method. Although this may be accounted for by the differences between arteriosclerotic vs. normal vessels or by interspecies differences, it is possible that the vascular endothelial cell coverage occurred earlier in the Microstent group because the stent was narrow and contained fewer stems. However, the issue of antiplatelet agents was considered to be a significant challenge to resolve for the future clinical application of the Microstent. We will have to consider the quantity and period of post-stent antiplatelet agents on the basis of the regimen after PCI in the clinical study. The quantity of medicine will decrease and the period of administration will be shortened more than the regimen after PCI, since the Microstent showed fewer biological response from the results of histology and SEM.

In the present experiments, most of the ischemic time was taken up by the procedure of fixing the adhesive, indicating that the operation time is also affected by this procedure. Since cyanoacrylate is an adhesive that is approved for in vivo usage, it was used in the present experiments to achieve the early clinical application of the Microstent. However, we believe that a further reduction in operation time, as well as an improvement in safety, can be attained by using new adhesives in future studies. By overcoming this issue, we believe that this NiTi stent (Microstent) is a device that can contribute significantly to the expansion of medical treatments that use microvascular anastomoses such as coronary artery bypass operations, microsurgeries of the tissues and organ transplantations. We have been developing another variant of Microstent for the end to side anastomosis as well as the end to end anastomosis, and at present, it is being evaluated by using a swine model.

## Conclusions

We were able to perform vascular anastomosis in a shorter amount of time than with the hand-sewn method by using a NiTi stent (Microstent) that we developed. Moreover, the Microstent method achieved similar or better results with regards to thrombus formation, lumen patency rate, and blood vessel strength in comparison to the hand-sewn method. We therefore believe that this NiTi stent (Microstent) is a device that can contribute significantly to the expansion of medical treatment that uses microvascular anastomosis.

## Supporting information

S1 Fig Computational fluid dynamics of a Microstent. (**A**) Generation of the luminal 3-D model of a vessel with a Microstent (arrowhead). To save time for the calculation, a half-pipe model was generated. The arrow indicates the direction of flow. (**B**) Meshing of the 3-D model around the stent branch junction by triangular prisms and trigonal pyramids. The surface of the 3-D model is covered by 2 layers of prisms with height of 7.5 μm, followed by pyramids. (**C-F**) Fluid stagnation around the junction of the stent branches is represented by a mean age of air (MAA) color chart. Microstents with a 100-μm-thick and regular junction curve (**C**), a 50-μm-thick and regular junction curve (**D**), a 100-μm-thick and narrowed junction curve (**E**), and a 150-μm-thick and regular junction curve (**F**) were analyzed. Red to yellow color represents significant stagnation of blood flow. Since the red colored area is significantly larger, it is suggested that thickness (**F**) and a narrowed junction curve (**E**) are related to higher MAA.
(TIF)

S1 Movie Surgical procedure of Microstent-assisted rat aorta anastomosis. A movie to show the intraoperative view of anastomosis using Microstent.
(MP4)

S2 Movie Ultrasonography of the rat aorta and Microstent at the anastomotic site. Longitudinal ultrasound view of rat aorta at the anastomotic site.
(MP4)

S3 Movie Burst strength test for hand-sewn vessels. A movie to demonstrate rupture of hand-sewn vessels under controlled pressure.
(MP4)

S4 Movie Burst strength test for Microstent-anastomosed vessels. A movie to demonstrate rupture of Microstent-anastomosed vessels under controlled pressure.
(MP4)

S5 Movie Tensile strength test for hand-sewn vessels. A movie to demonstrate break of hand-sewn vessels under controlled tension.
(MP4)

S6 Movie Tensile strength test for Microstent-anastomosed vessels. A movie to demonstrate break of Microstent-anastomosed vessels under controlled tension.
(MP4)
