# Supplementary figures and images for: Sutureless microvascular anastomosis assisted by an expandable shape-memory alloy stent
Source: PLoS One. 2017 Jul 24;12(7):e0181520. doi: 10.1371/journal.pone.0181520 (PMC5524395; doi:10.1371/journal.pone.0181520)

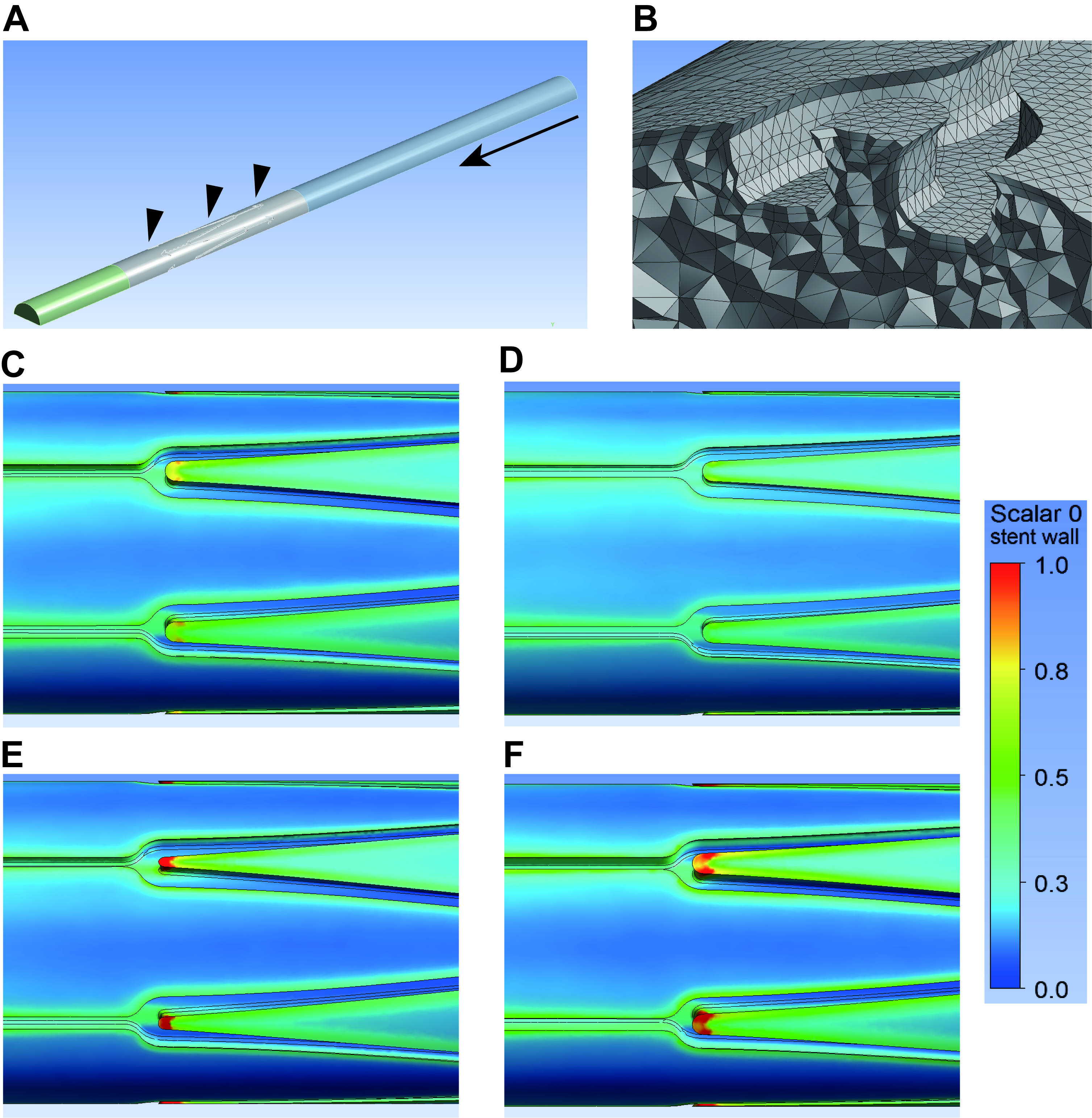

Supplement: S1 Fig — (A) Generation of the luminal 3-D model of a vessel with a Microstent (arrowhead). To save time for the calculation, a half-pipe model was generated. The arrow indicates the direction of flow. (B) Meshing of the 3-D model around the stent branch junction by triangular prisms and trigonal pyramids. The surface of the 3-D model is covered by 2 layers of prisms with height of 7.5 μm, followed by pyramids. (C-F) Fluid stagnation around the junction of the stent branches is represented by a mean age of air (MAA) color chart. Microstents with a 100-μm-thick and regular junction curve (C), a 50-μm-thick and regular junction curve (D), a 100-μm-thick and narrowed junction curve (E), and a 150-μm-thick and regular junction curve (F) were analyzed. Red to yellow color represents significant stagnation of blood flow. Since the red colored area is significantly larger, it is suggested that thickness (F) and a narrowed junction curve (E) are related to higher MAA. (TIF) [file pone.0181520.s001.tif]
